# An updated catalogue of diverse type II polyketide synthase biosynthetic gene clusters captured from large-scale nucleotide databases

**DOI:** 10.1099/mgen.0.000965

**Published:** 2023-03-23

**Authors:** Christina M. McBride, Eric L. Miller, Louise K. Charkoudian

**Affiliations:** ^1^​ Department of Chemistry, Haverford College, Haverford, PA, USA; ^2^​ Department of Biology, Haverford College, Haverford, PA, USA

**Keywords:** biosynthetic gene cluster, ketosynthase-chain length factor, polyketide synthase, type II polyketide

## Abstract

Nature serves as a rich source of molecules with immense chemical diversity. Aptly named, these ‘natural products’ boast a wide variety of environmental, medicinal and industrial applications. Type II polyketides, in particular, confer substantial medicinal benefits, including antibacterial, antifungal, anticancer and anti-inflammatory properties. These molecules are produced by enzyme assemblies known as type II polyketide synthases (PKSs), which use domains such as the ketosynthase chain-length factor and acyl carrier protein to produce polyketides with varying lengths, cyclization patterns and oxidation states. In this work, we use a novel bioinformatic workflow to identify biosynthetic gene clusters (BGCs) that code for the core type II PKS enzymes. This method does not rely on annotation and thus was able to unearth previously ‘hidden’ type II PKS BGCs. This work led us to identify over 6000 putative type II PKS BGCs spanning a diverse set of microbial phyla, nearly double those found in most recent studies. Notably, many of these newly identified BGCs were found in non-actinobacteria, which are relatively underexplored as sources of type II polyketides. Results from this work lay an important foundation for future bioprospecting and engineering efforts that will enable sustainable access to diverse and structurally complex molecules with medicinally relevant properties.

## Data Summary

All sequence data were obtained from the precompiled National Center for Biotechnology Information (NCBI) Basic Local Alignment Search Tool (BLAST) databases. The authors confirm all supporting data, code and protocols have been provided within the article or through supplementary data files.

**Impact Statement**

Nature – plants, bacteria, fungi and even animals – has the unique ability to produce molecules with a diverse range of structures and functions. Since many of these molecules have been successfully repurposed by humans in medical, environmental and industrial settings, developing strategies to identify the organisms and biosynthetic systems that manufacture natural products could help to find novel molecules of interest. Type II polyketides have unique antimicrobial and anticancer properties, so identifying new sources of these molecules may offer renewed access to promising pharmaceuticals. By conducting a global phylogenetic analysis of the chain-length factor (CLF) protein involved in type II polyketide biosynthesis, our work provides access to previously unexplored gene clusters that may harbour unique biodiversity. Our curated CLF set nearly doubled the number of sequences available to study, increasing both the volume and the diversity of potential polyketide synthases we can access *in vitro*.

## Introduction

Microbes have evolved the ability to manufacture a diverse profile of secondary metabolites that confer the host with a selective advantage over other organisms in the nearby environment [1, 2]. Humans have reaped the benefits of these molecules by repurposing secondary metabolites as key medicinal, environmental and industrial relevant agents. Type II polyketides, in particular, are celebrated for their potent medicinal applications, including as antibiotics (e.g. tetracycline) and anticancer agents (e.g. doxorubicin) [3]. These molecules are produced by type II polyketide synthases (PKSs), which are microbial protein assembly lines that iteratively assemble type II polyketides using discrete, monofunctional domains [4]. The minimal type II PKS – the simplest set of enzymes needed to facilitate biosynthesis – consists of three core domains. The ketosynthase (KS, or KSα) and chain length factor (CLF, or KSβ) exist as a heterodimer (namely, the KS–CLF) and drive polyketide elongation, while an acyl carrier protein (ACP) tethers and transports acetate-based building blocks and growing polyketide intermediates to and from each enzymatic domain [5, 6]. Once working in concert with additional tailoring enzymes, these type II PKSs are capable of rapidly producing molecules of immense complexity [5].

Microbial enzyme assemblies like these are encoded by biosynthetic gene clusters (BGCs), which are physically grouped genes that programme the production of a metabolite [7]. Previous studies have established bioinformatic methods to study the evolution of type II PKS BGCs, which has enabled the inferred history to influence future bioprospecting efforts [8–10]. Potential key duplication events in KS–CLF evolution were identified through the initial study, noting that type II KS–CLF BGCs may have diverged from the fatty acid synthase (FAS) KS, FabF, ancestor prior to the formation of the well-studied actinobacterial phylum [8, 10]. Most notably, this work highlighted the presence of type II PKS BGCs across a wide variety of non-actinobacteria [8]. Prior to this finding, research in the field was biased toward well-studied actinobacterial organisms [8], so shining light on these non-actinobacteria systems opens the way for exploration of new, previously untouched biosynthetic territory. Several non-actinobacteria type II PKS systems have been characterized, including BGCs from *Ktenobacter racemifer, Photorhabdus luminescens* TT01, *Streptoccocus* sp. GMD2S and *Pseudoalteromonas luteoviolacea *, revealing the possibility of innovation in this biosynthetic space [11, 12]. While a 2015 report ultimately identified 544 total putative type II PKS BGCs from all available nucleotide databases at the time of analysis [8], a 2022 inquiry reflects the rapid growth of deposited BGCs, reporting 3421 total putative type II PKS BGCs found within the annotated bacterial reference sequence genomes alone [9]. Analysis of the phylogeny of characterized clusters from this set highlighted how product class can overlay on the CLF evolutionary cladistics [9], further elucidating the previously noted connection to backbone chain length [8]. Ancestral non-oxidative PKSs that span several bacterial phyla deviate from this trend, which suggests an ancient ancestor of these genes evolved before the divergence of these multiple phyla [9].

With the ongoing growth of available genetic data [13], routinely updating bioinformatic searches like these serves as a roadmap to understand and explore type II PKS diversity. However, relying on annotated genetic data alone to identify these proteins of interest limits our access to unique environmental and uncultured samples. Here, we establish an updated bioinformatic pipeline to identify putative genes coding for the expression of KS and CLF proteins without the need for genome annotation. Using this pipeline, we identified 6352 KS–CLF pairs across 5552 nucleotide records flagged from our curated NCBI nucleotide databases, nearly doubling the available repository of type II PKS BGCs to explore. Further interpreting the evolutionary relationships between these clusters may offer us the information needed to understand these BGCs and their encoded synthases and flag regions which may be ripe for bioprospecting.

## Methods

### Nucleotide data

We built our nucleotide databases from the following precompiled NCBI blast databases [14] and locally compiled bacterial/archaeal NCBI reference sequence genomic databases:

- env_nt (metagenome/environmental nucleotide sequences; 15 June 2022),
- nt (nucleotide sequences from GenBank/EMBL/DDBJ; 15 June 2022),
- patnt (patent nucleotide sequences; 15 June 2022),
- tsa_nt (non-project based Transcriptome Shotgun Assembly entries; 15 June 2022),
- ref_prok_rep_genomes (Refseq representative prokaryotic genomes; 29 July 2022)

Therefore, our nucleotide databases consisted of complete genomes, contigs from draft genomes, contigs from environmental sequencing, transcriptome sequences and miscellaneous short nucleotide sequences.

### blast search for KS and CLF homologous sequences

These nucleotide databases were searched using translated nucleotide blast (tblastn) from the NCBI BLAST+ Suite v.2.11.0 for sequence homology to a set of known KS and CLF protein query sequences [15]. We selected proteins from the resistomycin, fredericamycin and WhiE spore pigment clusters as our query sequences, as these proteins have established sequence diversity among described type II PKS BGCs [8]. Significant alignments with an e-value ≤1 were processed; this threshold detected distantly related sequences, with spurious alignments removed in later stages of our analysis.

### Identifying open reading frames (ORFs) for each blast hit

We designed a custom Python script to search between 500 nt before and after a blast hit for the longest ORF that contains the blast hit. We considered a set of six possible start codons (ATG, CTG, GTG, TTG, ATT, ATC). An ORF was defined as the first of these start codons after a stop codon (or the edge of a given DNA sequence) and continuing until reaching an in-frame stop codon (or the edge of a given DNA sequence). By considering alternative start codons that are not ATG, our ORFs were perhaps longer on the 5′ end of the gene compared to the true start codon; however, our method makes the fewest assumptions when calling ORFs. We required that ORFs were at least 300 nt long and cover at least 50 % of the blast hit with an identical translated sequence, although the vast majority of hits matched 100 % of the blast hit. All custom Python scripts can be found at https://github.com/EricLMiller/KS_CLF_Search.

### Protein family classification via a profile hidden Markov model

Profile hidden Markov models (pHMMs) run on HMMER v. 3.3.2 were used to differentiate which KS and CLF ORFs belonged to type II PKS BGCs [16]. Predicted proteins resulting from translated ORFs were compared to standard KS and CLF sequence models established by Hillenmeyer *et al*. to best distinguish a given type II PKS protein from other protein families (e.g. fatty acid synthases or type I PKSs) or proteins within the same cluster (i.e. a type II KS vs. CLF) [8]. Sequence similarity score thresholds were selected based on the KS (score=352) and CLF (score=66) scores for the aurachin gene cluster, which is only distantly related to type II PKS systems and thus can be used as a cutoff to retain KS and CLF homologous hits [8, 17]. Translated ORFs with a sequence similarity score above or equal to the aurachin score thresholds were retained.

### Establishing close proximity of the KS and CLF genes

Using the information from the ORF-Finder on sequences that scored high in our pHMM, we wrote a custom Python script to find KS/CLF ORF pairs from the same nucleotide accession number. This script also removed pairs with over 2 kb distance between the genes to ensure that the KS and CLF function as a heterodimer and can be coexpressed, as noted by Hillenmeyer *et al*. [8]. KS/CLF matches that overlapped were included as passing this filter. Regions with more than two KS/CLF ORFs each within 2 kb of each other were all counted as possible, discrete KS/CLF pairs. All matches fitting these criteria with gene lengths less than 6 kb were retained.

### Phylogenetic analysis of KS and CLF genes

All multiple sequence alignments were produced using MAFFT Galaxy Version 7.475+galaxy0 [18]. Alignment sites with more than 5 % gaps across all sequences were removed to prevent undue influence of indel mutations. Maximum-likelihood phylogenetic trees were built using FastTree2 Galaxy Version 2.1.10+galaxy1 with the default JTT+CAT model, and internal nodes with less than 70 % bootstrap support were collapsed into polytomies [19]. We visualized the tree and associated data using ggtree v. 3.0.4 and ggtreeExtra v. 1.2.3 in R Studio [20–22]. We used the *Escherichia coli* FabF sequence WP_000044679.1 as an outgroup to root the tree.

### Further classification of KS/CLF hits

We utilized the Natural Product Domain Seeker version 2 (NaPDoS2), a phylogeny-based classification webtool, to predict KS and condensation domains, thus classifying our CLF hits [23]. A FASTA file containing the amino acid sequences of all relevant CLF hits was uploaded to the NaPDoS2 webpage (accessed 18 September 2022) and searched for KS domains using the default blastp e-value cutoff of 1×10^−8^ and minimum alignment length of 200 aa. We examined genomic regions 30 kb before and after each KS–CLF pair; multiple pairs within this range in single genomes were combined together. We ran antiSMASH v6.1.1 [24] using a minimum ORF length of 300 bp, prodigal v2.6.3 [25] as a gene-finding tool and ‘loose’ HMM-detection strictness. We searched the resulting antiSMASH .gbk files for gene_functions containing ‘t2ks’, ‘t2clf’ and ‘t2fas’. Additionally, the final CLF ORF set was compared to sets provided by Hillenmeyer *et al*. and Chen *et al*. [8, 9].

## Results

**Fig. 1.**
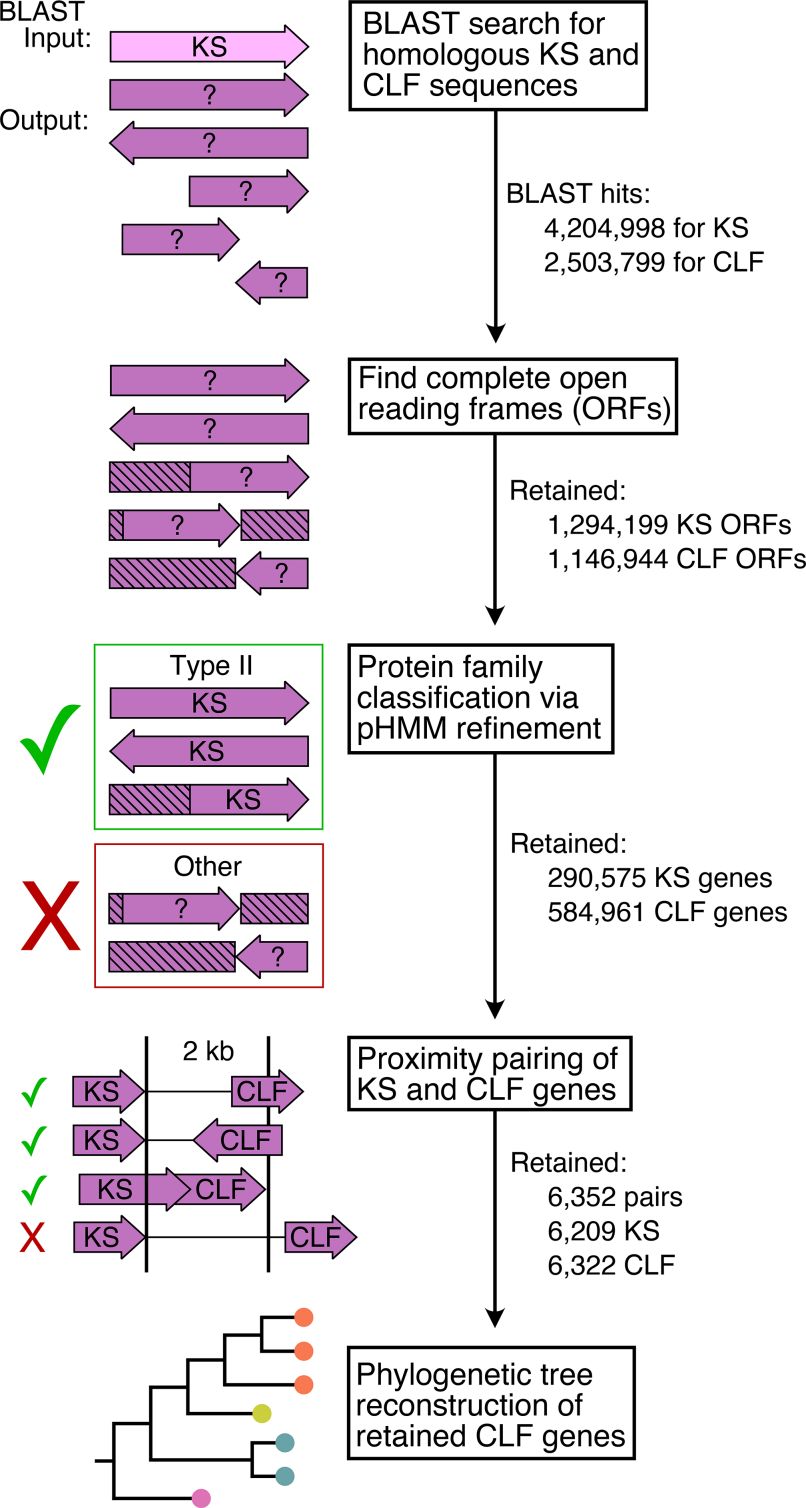
Bioinformatic pipeline to identify type II PKS KS–CLFs. Our workflow identified 6352 putative gene pairs coding for the expression of KS–CLFs across 14 phyla.

Using our pipeline (Fig. 1), we identified 6352 KS–CLF gene pairs across 5552 unique nucleotide records. These pairs consisted of 6209 KSs and 6322 CLFs; this discrepancy in the number of proteins arises from one KS being located in proximity to multiple CLFs, or vice versa.

We validated this pipeline by comparing the set of 167 characterized type II PKS CLFs reported by Chen *et al*. [9] with our 6322 CLF hits (Fig. S1, available in the online version of this article). Our curated CLF set contained 95.2 % of these characterized CLFs, with the following sequences missing in our analysis: five characterized CLFs that are no longer annotated on any genome available through NCBI; the dactylocycline A and thioangucycline CLFs reported by Chen, instead classifying these sequences as KSs; and the CLF for AQ-256, which was found using our blast-based search and retained based on our pHMM score but did not have a corresponding KS within 2000 bp and was thus removed during the proximity pairing stage. Since we found nearly all of the previously reported characterized type II PKS CLFs, and all the CLFs we would expect to find given our workflow, we are confident that our pipeline can accurately identify a broad range of CLF diversity.

**Fig. 2.**
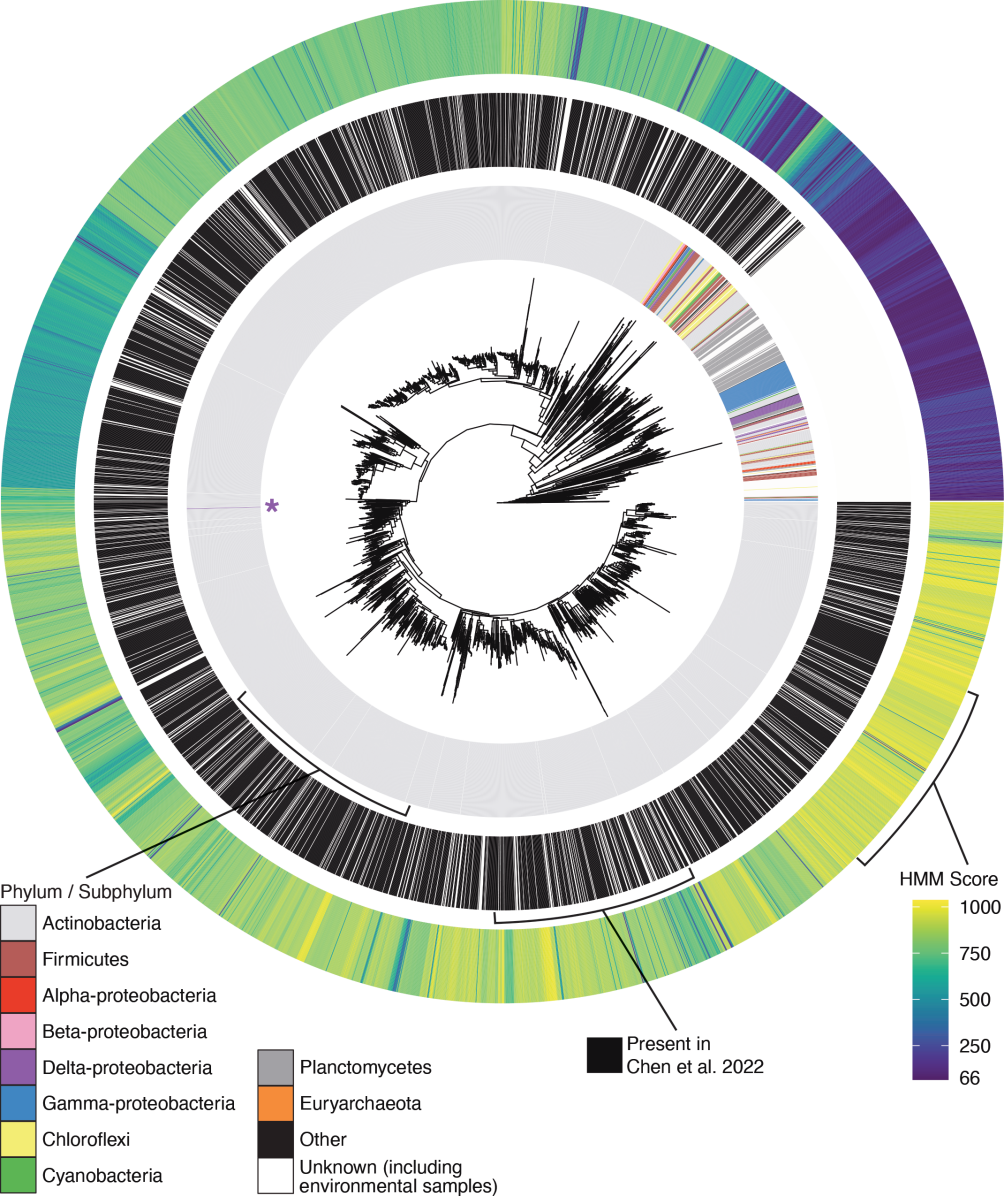
Phylogenetic tree representing 6322 type II PKS CLF protein sequences. The inner ring represents the phyla classification for the sequence, the middle ring signifies which sequences were also identified by Chen *et al*. in their recent publication, and the outer ring notes the pHMM score for each CLF amino acid sequence. The purple star indicates the location of a single non-actinobacteria hit (the deltaproteobacterium *Melittangeum boletus* DSM 14713) within the large actinobacteria clade. This phylogeny reveals a previously unexplored clade (indicated by the large white section of the middle ring) of primarily non-actinobacterial type II PKS CLFs.

Our curated KS–CLFs were found across both actinobacterial and non-actinobacterial prokaryotic species, representing, in total, 14 phyla: *Acidithiobacilla*, *Acidobacteria *, *Actinobacteria *, *Proteobacteria* (alpha, beta, delta and gamma), *Armatimonadetes *, *Candidatus Aminicenantes*, *Candidatus Cryosericota*, *Candidatus Omnitrophica*, *Chloroflexi *, *Cyanobacteria *, *Euryarchaeota *, *Firmicutes *, *Nitrospirae* and *Planctomycetes* (Fig. 2). Also included in this dataset were KS–CLFs identified from environmental samples and patented sequences. The environmental KS–CLF hits originated from both uncultured bacteria and various soil, bioreactor, groundwater, gut, human gut, hot spring, hydrothermal vent, marine, sediment, symbiont and wastewater metagenomes, illustrating the widespread prevalence of type II PKS enzymes. In total, 3380 of these CLF hits were also reported by Chen *et al*. in their 2022 study, meaning our remaining 2942 CLF hits were newly identified by our pipeline. Notably, we unearthed a large, non-actinobacterial clade not identified by Chen *et al*. (Fig. 2, middle circle).

To assess the significance of our data, we plotted the pHMM score for each CLF ORF onto the phylogeny, observing lower pHMM scores (corresponding to a weaker match to the selected model) for non-actinobacterial PKSs. The pHMM scores for actinobacterial hits had a median of 801.80 (with lower and upper quartiles of 747.80 and 893.70, respectively), while the pHMM scores for non-actinobacterial hits were much lower with a median of 132.25 (with lower and upper quartiles of 87.00 and 194.85, respectively). Since the pHMM score of the aurachin CLF was 66, these clusters score better against the model than the most distant type II BGC included in our tree, suggesting that these lower scores do not necessarily indicate that they are not true type II CLFs. As a result, to further verify the soundness of our CLF set, we used NaPDoS2 to detect and classify KS and CLF domains from our dataset. Ultimately, the NaPDoS2 algorithm identified 5497 type II CLF domains, 181 type II KS domains, 464 type II FAS domains, and 22 KS or condensation domains from other synthases (type II polyene, type II aryl polyene and type I modular *cis*-acyl transferase). In total, 158 of our CLF sequences were not classified.

**Fig. 3.**
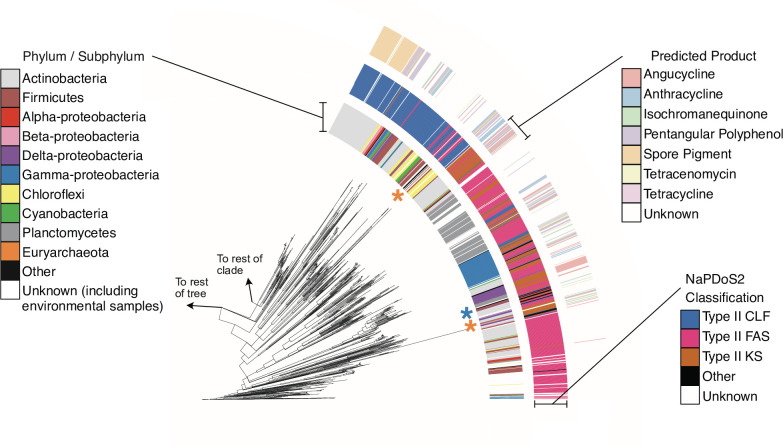
Subset of a phylogenetic tree representing the primarily non-actinobacteria clade of type II PKS CLF proteins. The inner ring represents the taxonomic classification, the middle ring identifies how the sequences were classified by NaPDoS2, and the outer ring represents the predicted molecular product type per NaPDoS2. The orange stars represent the locations of our archaeal hits, while the blue star represents the aurachin gene cluster chosen as the cutoff for similarity to type II PKS BGCs. The existence of type II PKS KS and CLF hits within this paraphyletic group closer to the root (as determined by outgroup *E. coli* FabF sequence WP_000044679.1) confirms that this previously unexplored region could represent an evolutionarly distinct subset of type II PKS BGCs, rather than false positive hits.

We continued our analysis by focusing on the non-actinobacterial sequences identified by our pipeline (Fig. 3). For accuracy of phyla determination, we excluded environmental samples and patented sequences from these analyses. Of the 565 non-actinobacterial CLF hits, NaPDoS2 classified 163 hits as type II CLFs, 107 hits as type II KSs, 263 hits as type II FAS KS domains, and 12 KS or condensation domains from other synthases (type II polyene or type II aryl polyene). Twenty of these non-actinobacterial CLF sequences were not classified. Of those sequences classified as type II KSs or CLFs, only 145 had predicted product types. Interestingly, whereas the large actinobacterial clade showed a connection between product type and phylogenetic similarity, we did not observe evidence of this association for the non-actinobacterial CLF sequences (Fig. S2).

While NaPDoS2 classified some of the hits within this previously unexplored non-actinobacterial clade as type II CLFs, others were instead classified as type II FAS KSs, type II KSs, other condensation domains or even unknown proteins. To further understand these classifications, we utilized antiSMASH to look for type II condensation domains in our nucleotide records of interest. As described in Table 1, we identified the presence of three different condensation domain pairings: (1) type II KS and type II CLF, (2) type II KS and type II FAS, and (3) two type II FASs. The most traditional type II PKS would be represented by those containing the type II KS and type II CLFs, which occurred in 5267 actinobacteria pairs and 87 non-actinobacteria pairs. However, the type II KS and type II FAS pairing (233 pairs in actinobacteria, 94 pairs in non-actinobacteria) and the type II FAS – FAS pairing (196 pairs in actinobacteria, 319 pairs in non-actinobacteria) have a significant presence in our curated CLF set. The existence of these different pairings of condensation domains may indicate that our pipeline captured CLF sequence diversity extending beyond the traditional understanding of the differences between type II PKS and type II FAS systems.

**Table 1.** antiSMASH-identified genes within antiSMASH-detected BGCs within 30 kb of our KS/CLF pairs

	**Type II KS and CLF**	**Type II KS and FAS**	**Type II FAS and FAS**
**Actinobacteria**			
KS–CLF pairs (5348 total)	5267 (98.5 %)	233 (4.4 %)	196 (3.7 %)
Unique GenBank accessions* (4862 total)	4799 (98.7 %)	232 (4.8 %)	196 (4.0 %)
**Non-actinobacteria**			
KS–CLF pairs (546 total)	87 (15.9 %)	94 (17.2 %)	319 (58.4 %)
Unique GenBank accessions* (501 total)	87 (17.4 %)	91 (18.2 %)	315 (62.9 %)

*In total, 18.6 % of actinobacteria accessions and 3.0 % of non-actinobacteria accession numbers have multiple regions >30 kb apart, each with KS–CLF pairs, indicating the presence of multiple type II PKS BGCs within the same nucleotide record.

## Discussion

Our bioinformatic pipeline identified 6322 individual CLF hits, nearly double those found by Chen *et al*. in April 2022 and around 11 times larger than the set reported in 2015 [8, 9], confirming the repeated need for updated bioinformatic efforts as the nucleotide databases grow. The variation of phyla represented in the tree exceeds that of previous searches (ours represented 14 phyla, while Chen *et al*. had eight and Hillenmeyer *et al*. had ten), opening up routes for new type II polyketide biosynthetic diversity outside of the traditional actinobacterial space. Notably, we identified a primarily non-actinobacterial clade that has not been previously identified in the literature. Past bioinformatic searches have utilized a variety of criteria (Pfam domain annotation, pHMM searches, antiSMASH BGC classification) with varying stringency, so it is unsurprising that these different methods produce different sequence sets. Our model may be less stringent than recent searches, leading to a broader selection of CLFs that could represent more distantly evolved, biosynthetically unique BGCs.

It is important to highlight the archaeal KS–CLF hits identified by our pipeline. Previously, modern archaea and their last common ancestor have been shown to contain FAS pathways, but these archaeal systems lack an ACP [26]. More recently, a survey of modular enzymatic synthase BGCs – which produce similar small molecule products to type II PKSs – was performed across bacterial, archaeal and eukaryotic genomes. Three archaeal non-ribosomal peptide synthetase (NRPS) pathways were identified in strains of *Methanobacteria* and *Methanomicrobia* but no type I PKS BGCs were found in archaea, suggesting that these small-molecule-producing clusters are elusive and rare [27]. Our pipeline ultimately reported three CLFs from *Euryarchaeota,* all members of the *Candidatus Methanoperedenaceae* archaeon species. While NaPDoS2 classified one of these CLF hits as belonging to a type II FAS cluster, the other two hits were classified as type II PKS KS proteins, posing the question as to whether archaea may actually be capable of producing polyketide-like products.

Mirroring the results of recent phylogenetic analyses [9], the deltaproteobacterium *Melittangeum boletus* DSM 14713 was the only non-actinobacterial CLF found within a large, otherwise actinobacterial clade. Our results are consistent with a single large horizontal gene transfer (HGT) event, as suggested by Chen *et al*. [9].

As a result of their relative evolutionary separation, there are distinct differences between the various phyla represented in our tree. On average, the actinobacterial CLFs scored substantially higher in our pHMM model than the non-actinobacterial CLFs. To some extent, this discrepancy indicates that the non-actinobacterial CLFs are less similar to known, characterized CLF proteins. However, since all of the CLF proteins used to build the model were from actinobacteria, this lower score may actually reflect the current bias in the field toward actinobacterial type II PKSs.

To further verify our CLF hits, we ran all 6322 CLF sequences through NaPDoS2, a phylogeny-powered webtool that classifies various KS and condensation domains [23]. Approximately 95 % of our actinobacterial CLF hits were classified as type II PKS CLF domains by NaPDoS2, verifying the relative accuracy of our pipeline. On the other hand, only 29 % of our non-actinobacterial CLF hits were classified as type II PKS CLF domains via NaPDoS2, whereas 48 % were classified as type II FAS, 17 % were classified as type II KS, 2 % were classified as some other KS domain and the remaining 5 % were not classified. Most of the non-actinobacterial hits classified as type II CLFs have a closer evolutionary relationship to actinobacterial CLFs, indicating that the characteristic sequence motifs of CLF proteins are dictated by those found across actinobacterial CLFs. Nonetheless, this presence of CLFs across both actinobacterial and non-actinobacterial phyla supports the inference that the ancient FAS *ks* gene that was duplicated to form the type II PKS KS and CLF genes originated prior to the most recent common ancestor of the actinobacteria and of *E. coli *, whose FAS is used as an outgroup for these genes [8, 10].

While the CLF proteins with closer homology to the *E. coli* FAS KS were less likely to be classified as type II PKS CLFs by NaPDoS2, this does not mean that these hits are extraneous sequences accidentally retained by our pipeline as a previously unexplored clade. Many of these CLF hits were classified as either type II FAS or type II KS domains by NaPDoS2; since our workflow mandated the presence of a KS and CLF gene within 2000 bp of each other to retain them as a KS–CLF pair, each of these hits must have another KS-like domain within these bounds. Therefore, these seemingly extraneous hits could represent BGCs that evolved directly after the ancient FAS *ks* gene duplication and thus have two near-identical KS domains that lack the typical motifs seen across most actinobacterial CLFs. Despite their relative infrequency, there were CLF hits within this previously unexplored clade that were classified as type II CLF genes, indicating that our workflow is capturing distant CLF genes that may harbour unique sequence and biosynthetic diversity.

Our antiSMASH analysis of the regions surrounding our hits further supports this inference. While 15.9 % of our non-actinobacterial hits had the traditional type II KS – type II CLF pairing, 17.2 % of these examined regions contained type II KS – type II FAS pairs and 58.4 % of these regions contained type II FAS – type II FAS pairs. Differences between the percentage of actinobacteria and non-actinobacteria pairs classified as type II KS/type II CLF are hard to interpret due to the actinobacteria sequenced at a much high phylogenetic density compared to the non-actinobacteria; genomes are often not randomly selected to be sequenced, and so the resulting collection of genomic information in NCBI can be biased accordingly. The type II KS – type II FAS pairing represents a relatively unexplored region of BGC diversity. Hits of this type contain two condensation domains: one is homologous to known type II KSs, while the other is most similar to known type II FAS KS domains. This pairing may indicate that hybrid type II PKS/type II FAS systems exist, and that our definition of what constitutes a ‘CLF’ may need to be broadened, such that type II CLFs may actually look much more similar to type II FAS KSs than we previously understood. We also note several non-canonical clusterings of three condensation domains, such as type II KS/type II FAS/type II CLF or type II KS/type II CLF/type II CLF triads. Further characterizing atypical BGC types may reveal biosynthetic diversity beyond our current understanding of type II PKS and type II FAS systems. While the type II FAS – type II FAS pairs may just represent regions where the FAS KS gene duplicated and thus still belong to a traditional type II FAS system, these clusters still represent an area of biosynthetic diversity that should be explored. Delineating the differences between these pairs of condensation enzymes may provide further insight into what constitutes a type II PKS vs. a type II FAS and how we might manipulate these systems to allow them to combinatorially interact.

Our bioinformatic pipeline, developed using the computational resources made available through a primarily undergraduate institution, can be easily understood and manipulated by researchers with limited computational experience. While initially designed to identify type II PKS KS–CLF pairs, our pipeline is not unique to type II PKS systems; rather, each step can be tailored to incorporate the parameters needed to produce a phylogeny of any evolutionarily conserved gene or protein, such as by changing the reference set used for comparison in the pHMM or changing the conditions for gene length or proximity. Since our pipeline does not rely on previous annotation, users can systematically identify homologues of their sequence of interest from sequence data of varying completeness or accuracy.

## Conclusions

Unveiling new routes to type II polyketide-like molecules could offer swift access to promising bioactivity. Using the bioinformatic pipeline described herein, we report an updated profile of CLF diversity – a critical and unique protein to type II PKSs – across 14 distinct microbial phyla. Along with unveiling a previously undescribed, primarily non-actinobacterial clade, we share potential evidence of type II polyketide biosynthetic potential in archaea. We further provide additional support for the inference that the type II KS and CLF genes diverged from a common ancestor of the *E. coli* KS prior to the formation of the actinobacterial clade.

The curated CLF set itself serves as a ‘biosynthetic catalogue’, offering insight into potential leads for type II PKS bioprospecting efforts. Exploring the previously undescribed non-actinobacterial clade may unveil new type II PKS enzymatic and molecular diversity, guiding future efforts in the biosynthesis of never-before-seen secondary metabolites. Further understanding the existence of this clade may allow us to dissect the differences between type II FAS and PKS KS-like domains, gaining insight into the divergent evolution of these systems. We share these results with the community with the hope that they will spark efforts to characterize these diverse BGCs and encoded enzymes. Uncovering the biosynthetic prowess of these systems represents a critical step in gaining sustainable access to important molecules.

## Supplementary Data

Supplementary material 1

Supplementary material 2
