# Corrigendum: An updated catalogue of diverse type II polyketide synthase biosynthetic gene clusters captured from large-scale nucleotide databases

**DOI:** 10.1099/mgen.0.001348

**Published:** 2025-02-11

**Authors:** Christina M. McBride, Eric L. Miller, Louise K. Charkoudian

**Affiliations:** 1 Department of Chemistry, Haverford College, Haverford, PA, USA; 2 Department of Biology, Haverford College, Haverford, PA, USA

In the published version of this article, there was a minor error in the text under the ‘Results’ section of the article.

The text appears as follows:

‘Our curated CLF set contained 95.2% of these characterized CLFs, with the following sequences missing in our analysis: five characterized CLFs that are no longer annotated on any genome available through NCBI; the dactylocycline A and thioangucycline CLFs reported by Chen, instead classifying these sequences as KSs; and the CLF for AQ-256, which was found using our blast-based search and retained based on our pHMM score but did not have a corresponding KS within 2000 bp and was thus removed during the proximity pairing stage. Since we found nearly all of the previously reported characterized type II PKS CLFs, and all the CLFs we would expect to find given our workflow, we are confident that our pipeline can accurately identify a broad range of CLF diversity.’

This misstates the reason why the CLF for AQ-256 was not retained. The correct text should be:

Our curated CLF set contained 95.2% of these characterized CLFs, with the following sequences missing in our analysis: five characterized CLFs that are no longer annotated on any genome available through NCBI; the dactylocycline A and thioangucycline CLFs reported by Chen, instead classifying these sequences as KSs; and the CLF for AQ-256, which was found using our blast-based search **but not retained based on a pHMM score of 61.3, thereby below our threshold score of 66**. Since we found nearly all of the previously reported characterized type II PKS CLFs, and all the CLFs we would expect to find given our workflow, we are confident that our pipeline can accurately identify a broad range of CLF diversity, **although there are lingering questions about the exact recommended pHMM threshold or if a pHMM is the most robust approach**.

In addition, Fig. S1 has been amended to reflect the textual change. In the original Fig. S1 in the supplementary PDF, three CLFs (from the AQ-256, julichrome, and cosmomycin C biosynthetic gene clusters) were mistakenly labelled in the phylogenetic tree. The labels for these three CLFs have now been removed from the tree, and the remaining labels were manually inspected to ensure that the CLFs were accurately represented in both the phylogeny and the curated dataset.

**Figure:**
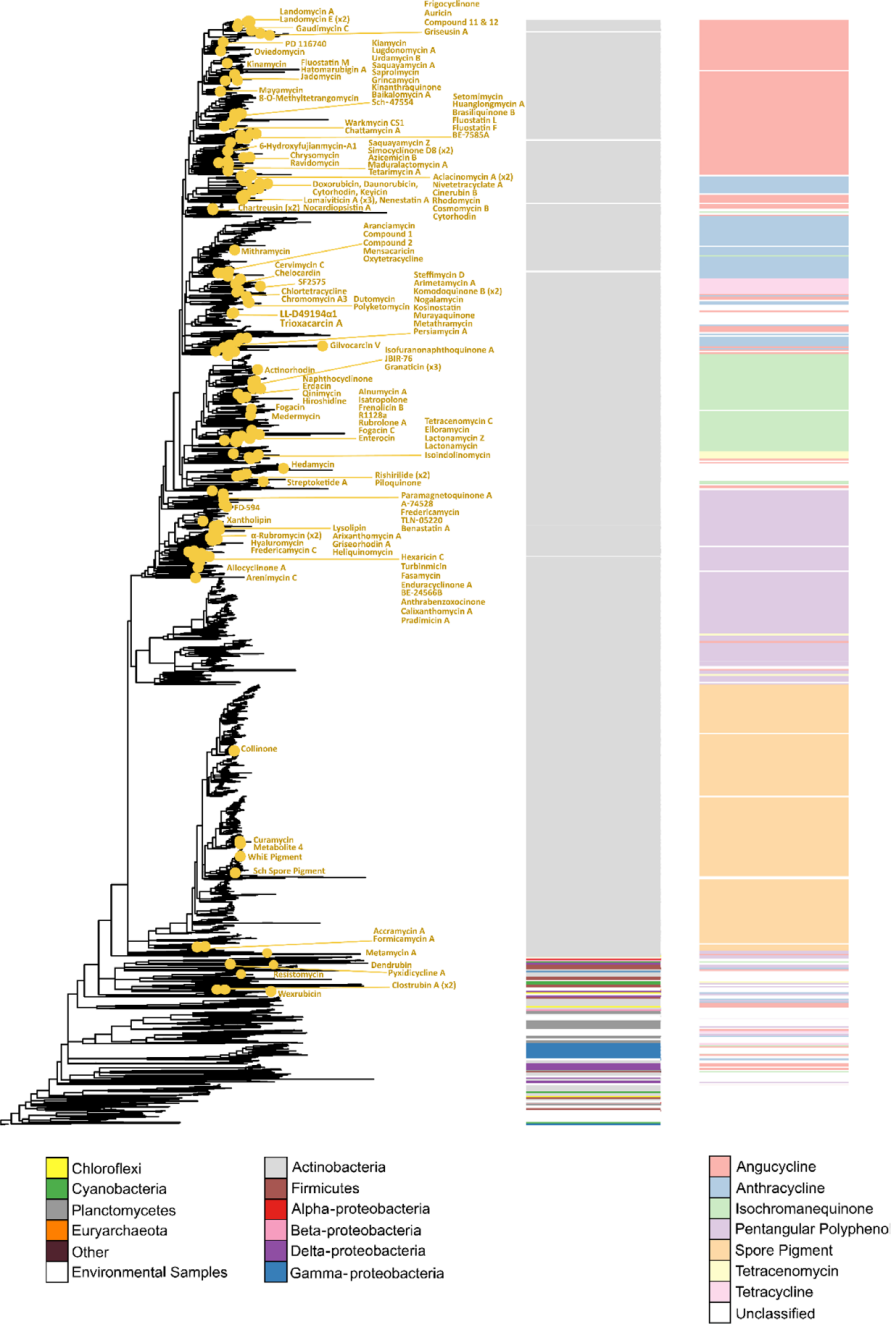
**Figure S1.** Phylogenetic tree representing 6,322 type II PKS CLF protein sequences. The yellow dots mark the position of CLFs that match a known, characterized CLF sequence as provided by Chen *et al* (2022). Since nearly all of the 167 characterized CLFs were represented in this tree, we are confident that our workflow can successfully identify CLF diversity across a variety of clades.

The authors apologise for any inconvenience caused.

