# Supplementary material for: An updated catalogue of diverse type II polyketide synthase biosynthetic gene clusters captured from large-scale nucleotide databases
Source: Microb Genom. 2023 Mar 23;9(3):mgen000965. doi: 10.1099/mgen.0.000965 (PMC10132072; doi:10.1099/mgen.0.000965)
Supplement: Supplementary material 1 [file mgen-9-965-s001.pdf]

Supporting Information

An updated catalog of diverse type II polyketide synthase biosynthetic gene clusters captured from large-scale nucleotide databases

Christina M. McBride[1], Eric L. Miller[2], and Louise K. Charkoudian[1]

[1] Department of Chemistry, Haverford College, Haverford, PA, USA.
[2] Department of Biology, Haverford College, Haverford, PA, USA.

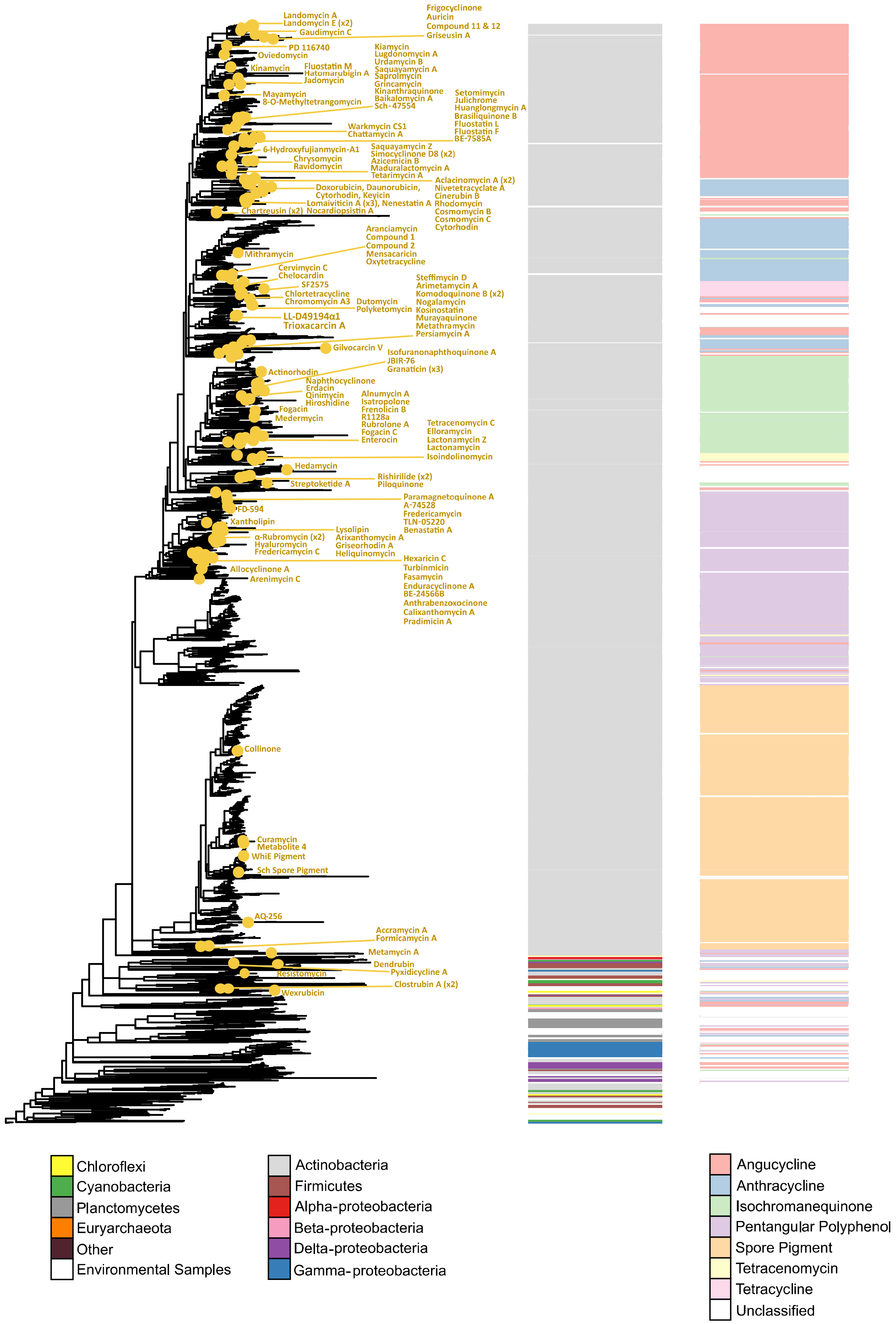

Figure S1. Phylogenetic tree representing 6,322 type II PKS CLF protein sequences. The yellow dots mark the position of CLFs that match a known, characterized CLF sequence as provided by Chen et al (2022). Since nearly all of the 167 characterized CLFs were represented in this tree, we are confident that our workflow can successfully identify CLF diversity across a variety of clades.

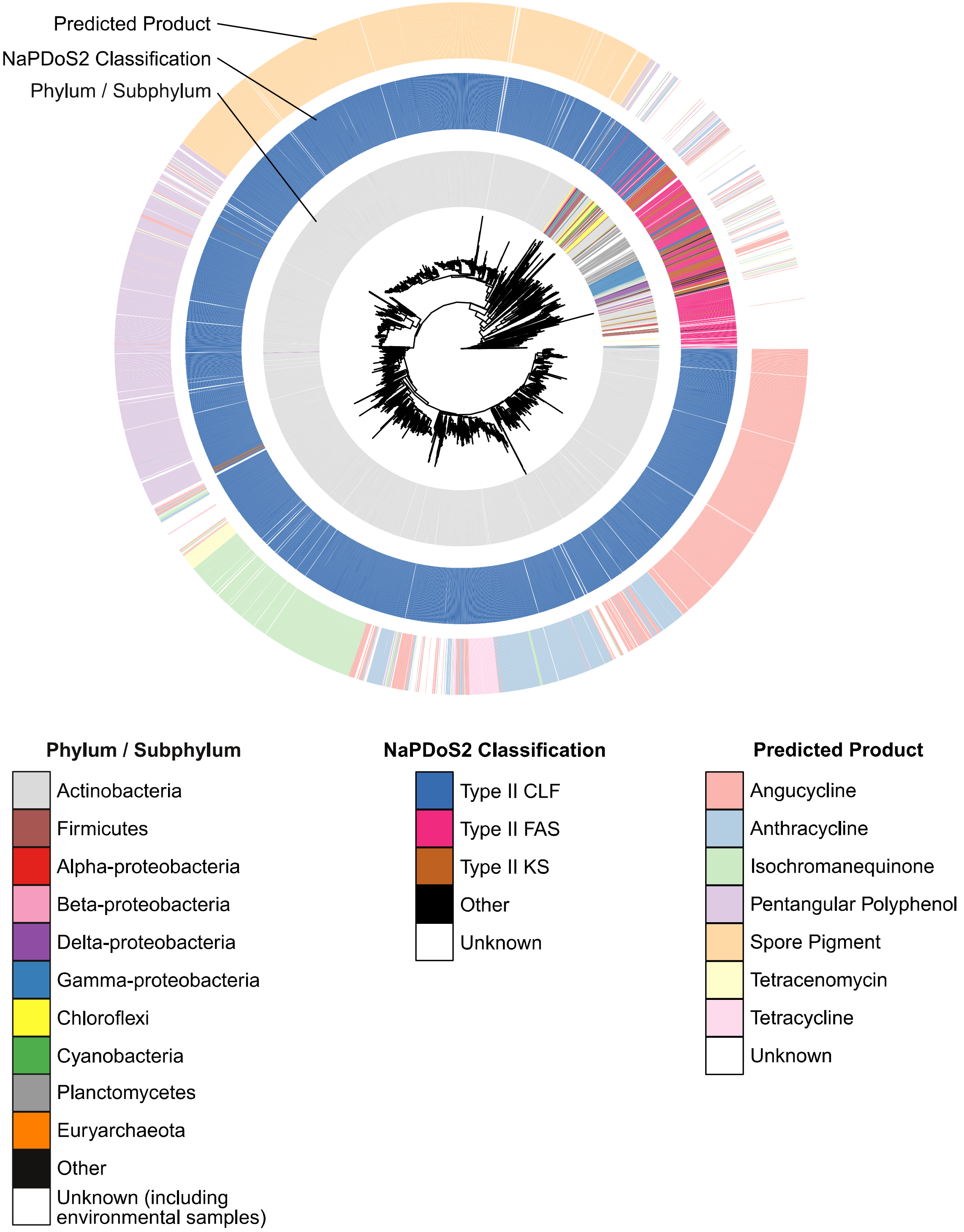

Figure S2. Phylogenetic tree representing 6,322 type II PKS CLF protein sequences. The inner ring represents the taxonomic classification, the middle ring identifies how the sequences were classified by NaPDoS2, and the outer ring represents the predicted molecular product type per NaPDoS2. We observe that the predicted product type is grouped by clade in Actinomycetes, while the non-Actinomycete clade products are largely unexplored.
